# Correction: Emergence of Slow-Switching Assemblies in Structured Neuronal Networks

**DOI:** 10.1371/journal.pcbi.1005288

**Published:** 2017-02-10

**Authors:** The PLOS Computational Biology Staff

An error occurred during the typesetting process. Some of the reference citations are incorrect.

In the following places, reference [29] is corrected to [27]:

At the end of Figure 1, in the Introduction.

At the end of paragraph three, in the Results section.

At the end of paragraph one, under the subheading ‘Linear rate models and slow localized activity in clustered LIF networks’, in the Results section.

In the middle of paragraph four, under the subheading ‘Linear rate models and slow localized activity in clustered LIF networks’, in the Results section.

At the end of paragraph four, under the subheading ‘Linear rate models and slow localized activity in clustered LIF networks’, in the Results section.

At the end of paragraph five under the subheading ‘Linear rate models and slow localized activity in clustered LIF networks’, in the Results section.

At the beginning of paragraph one, under the subheading ‘The linear rate model and the full dynamics of clustered LIF networks’, in the Results section.

At the beginning of paragraph one, under the subheading ‘The block-localization of the dominant linear subspace’, in the Results section.

After Equation (12), under the subheading ‘Beyond clustered excitatory neurons: SSA dynamics involving both excitatory and inhibitory neurons’, in the Results section.

After Equation (13), under the subheading ‘Beyond clustered excitatory neurons: SSA dynamics involving both excitatory and inhibitory neurons’, in the Results section.

At the end of paragraph three, in the Discussion section.

At the end of paragraph six, in the Discussion section.

In the following place, reference [28] is corrected to [27]:

At the beginning of paragraph four under the subheading, ‘A stylized linear rate model for networks with clustered excitatory neurons’, in the Results section.

The publisher apologizes for this error. The correct references can be viewed in the preprint version of the article: https://arxiv.org/abs/1502.05656
