# Supplementary figures and images for: Emergence of Slow-Switching Assemblies in Structured Neuronal Networks
Source: PLoS Comput Biol. 2015 Jul 15;11(7):e1004196. doi: 10.1371/journal.pcbi.1004196 (PMC4503787; doi:10.1371/journal.pcbi.1004196)

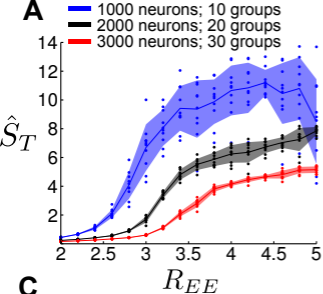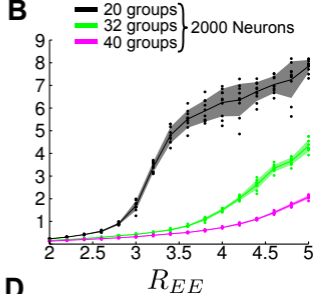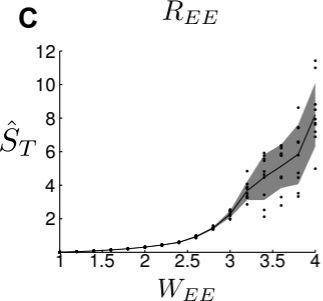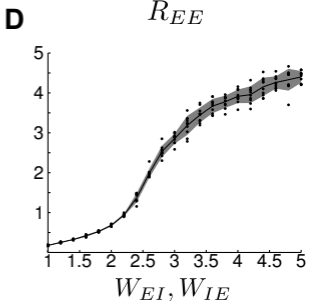

Supplement: S1 Fig — A Clustering Strength R EE vs spike rate variability across time S^T for varying network sizes with a fixed assembly size. Compare to Fig 2A of the main text. B Clustering Strength R EE vs spike rate variability across time S^T for a fixed network size with varying assembly sizes. Compare to Fig 2B of the main text. C Weight Clustering W EE vs spike rate variability across time S^T for a LIF network with 2000 neurons (20 groups; topological clustering R EE = 1). Compare to Fig 4 of the main text. D Spike rate variability across time S^T as a function of W EI = W IE for a network with excitatory to inhibitory feedback loops. Compare to Fig 8 of the main text. Dots: raw data from simulations; line: mean, shading: standard deviation. (PDF) [file pcbi.1004196.s001.pdf]

**A**

Scale free network

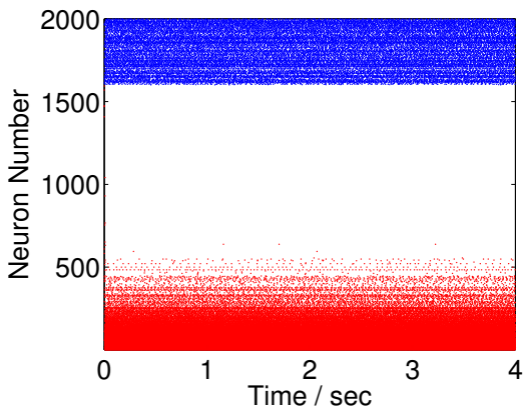**B**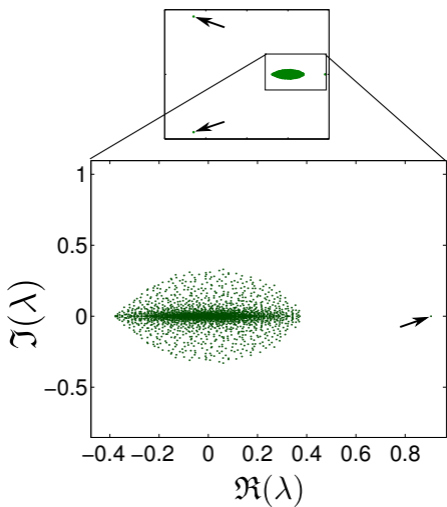

Supplement: S2 Fig — A Raster plot for an example scale-free network. Excitatory neurons are ordered according to their (expected) degree: high (bottom) to low (top). Note that the spiking activity is largely concentrated around the hub (which due to assortativity is connected to other nodes of high degree), and there are no distinguishable groups or switching behavior. B Spectrum of the simulated scale-free network. There is only one eigenvalue separated from the main bulk of the spectrum due to the presence of the large hub in the network. (PDF) [file pcbi.1004196.s002.pdf]
